# Decreasing trend in preterm birth and perinatal mortality, do disparities also decline?

**DOI:** 10.1186/s12889-020-08925-w

**Published:** 2020-05-26

**Authors:** Anita C. J. Ravelli, Martine Eskes, Joris A. M. van der Post, Ameen Abu-Hanna, Christianne J. M. de Groot

**Affiliations:** 1 Department of Medical Informatics, Amsterdam UMC, University of Amsterdam, Amsterdam, The Netherlands; 2 Department of Obstetrics and Gynaecology, Amsterdam UMC, University of Amsterdam, Amsterdam, The Netherlands

**Keywords:** Perinatal mortality, Preterm birth, Trends, Population based cohort, Health inequalities, small for gestational age, maternal age, ethnicity, risk group analysis, socio-economic status.

## Abstract

**Background:**

In the Netherlands, several initiatives started after the publication of the PERISTAT findings that showed the perinatal mortality risk was higher than in other European countries. The objective of this study is 1) to report recent trends in perinatal mortality and in intermediate risk groups (preterm birth, congenital anomalies and small for gestational age (SGA)), 2) describing perinatal mortality risk among children born preterm, with congenital anomalies or SGA, and born in maternal high risk groups (parity, age, ethnicity and socio-economic status (SES)).

**Methods:**

A nationwide cohort study in the Netherlands among 996,423 singleton births in 2010–2015 with a gestational age between 24.0 and 42.6 weeks. Trend tests, univariate and multivariable logistic regression analyses were used. We did separate analyses for gestational age subgroups and line of care.

**Results:**

The perinatal mortality rate was 5.0 per 1000 and it decreased significantly from 5.6 in 2010 to 4.6 per 1000 in 2015. Preterm birth significantly declined (6.1% in 2010 to 5.6% in 2015). Analysis by gestational age groups showed that the largest decline in perinatal mortality of 32% was seen at 24–27 weeks of gestation where the risk declined from 497 to 339 per 1000. At term, the decline was 23% from 2.2 to 1.7 per 1000. The smallest decline was 3% between 32 and 36 weeks.

In children with preterm birth, congenital anomalies or SGA, the perinatal mortality risk significantly declined. Main risk factors for perinatal mortality were African ethnicity (adjusted odds ratio (aOR) 2.1 95%CI [1.9–2.4]), maternal age ≥ 40 years (aOR1.9 95%CI [1.7–2.2]) and parity 2^+^ (aOR 1.4 95%CI [1.3–1.5]). Among the (post)term born neonates, there was no significant decline in perinatal mortality in women with low age, low or high SES, non-Western ethnicity and among women who started or delivered under primary care.

**Conclusions:**

There is a decline in preterm birth and in perinatal mortality between 2010 and 2015. The decline in perinatal mortality is both in stillbirths and in neonatal mortality, most prominently among 24–27 weeks and among (post)term births. A possible future target could be deliveries among 32–36 weeks, women with high maternal age or non-Western ethnicity.

## Background

It’s important to monitor perinatal health indicators including perinatal mortality and preterm birth. These are key indicators of quality of care in pregnancy and childbirth. The EURO-Peristat project showed that stillbirth and neonatal mortality rates from 2004 declined over all Europe but mortality disparities between the European countries still exist [1, 2]. In 2010 in the Netherlands, stillbirth and neonatal mortality were high compared to other European countries [2–4]. In 2015 the perinatal mortality rates further declined with still high heterogeneity between the European countries, all described in the new PERISTAT report [5]. Preterm birth (birth < 37 weeks of gestation) accounts for 75% of the perinatal mortality cases [3]. Preterm birth, congenital anomalies and/or small for gestational age (SGA p10) are the main intermediate risk group for perinatal mortality, also called “the Big 3” [6, 7].

In the Netherlands, several initiatives started to reduce the perinatal mortality rate. In 2010 active management for extremely preterm births at 24 weeks instead of at 25–26 weeks onwards began [8]. Also in 2010 a national perinatal audit program on term perinatal mortality started and within two years all 82 perinatal health care regions (hospital(s) and their surrounding primary care midwifery practices that are their preferred referring practices) participate in the national audit programme [9]. The ministry of health also made it possible that the College for Perinatal Care (CPZ) could be established in which all relevant partners in perinatal care participate. Perinatal health care regions have investigated in new cooperation forms like integrated obstetric care [10]. In addition, in the Netherlands a structural ultrasound scan at 20 weeks has been introduced, which provided before 24 weeks more diagnoses and possible terminations of pregnancies. This resulted in a reduction in serious congenital abnormalities after 24 weeks [11]. Pregnant women with gestational age > 41 weeks are earlier referred from a midwife to an obstetrician for foetal condition assessment and possible induction of labour [12–14]. To improve perinatal health inequalities “Healthy pregnancy for all” programmes were funded for several municipalities with increased perinatal mortality risk [15–17].

Main known risk factors for perinatal mortality are teenage pregnancy and advanced maternal age [18], smoking, non-Western ethnicity [19], socio-economic disadvantage [20], maternal obesity and complicated pregnancy history for instance preterm birth [3, 8]. Women in Europe living under adverse socio-economic circumstances have twice the perinatal mortality risk. Stillbirth, neonatal mortality and preterm births are major public health priorities with significant disparities based on socio-economic status and ethnicity and these disparities are persistent [21, 22]. Reduction of perinatal mortality rates can be targeted at each risk group and information on these effects are not yet known [23].

The objective of this study is to answer the following questions;

- What is the recent trend in perinatal mortality (stillbirth, neonatal) and in the intermediate risk groups (preterm birth, congenital anomalies, small for gestational age (SGAp10)) from 2010 onwards?
- In which intermediate risk group (preterm, congenital anomalies, SGA) or main risk group (parity, maternal age, socio-economic status and ethnicity) or line of care is the largest perinatal mortality reduction seen and where is still opportunity for improvement?

## Methods

This study was based on data from the national perinatal registry (PERINED) in The Netherlands [14]. Participation in the registry is obligatory for midwives, obstetricians and neonatologists practices, and in the recent years 98% of all births in the Netherlands are included in the registry [24]. Data includes information about pregnancy, child birth and admission of the child within one month after birth [14, 25].

We selected a population based cohort of all 996,423 singletons born between 24.0 and 42.6 weeks of gestation in a six year period (Jan 1, 2010 up to and including Dec 31, 2015). As in the PERISTAT analysis we excluded births at 22 and 23 weeks of gestation. The data in the perinatal registry are anonymised. The PERINED committee for research and ethics approved the use of data for this overall trend study (Approval number 16.13).

### Main outcome measurements

Perinatal mortality consists of stillbirth (death of a child before or during delivery) and neonatal mortality (death of a live born in the first 4 weeks of life). Perinatal mortality rate was defined from 24 weeks onwards as stillbirth or neonatal mortality within 28 days after birth per 1000 births. The term stillbirth is interchangeably used for foetal mortality.

### Intermediate risk group factors

We looked at the following intermediate risk group factors for perinatal mortality; preterm birth (gestational age < 37 weeks) and gestational age in six different gestational subgroups: (24.0–27.6, 28.0–31.6, 32.0–36.6, 37.0–38.6, 39.0–40.6, 41.0–42.6 weeks of gestation), congenital anomalies (yes/no) and small for gestational age < p10 (SGA p10) (yes/no) [3]. Small for gestational age was defined as below the 10th centile adjusted for parity, gestational age, gender and ethnicity [26].

### Risk factors

The following risk factors for perinatal mortality were analysed: parity (0, 1, 2 or higher), maternal age (< 20, 20–24, 25–34, 35–39, ≥40), socio-economic status (low <p25, mid, high ≥p75), maternal ethnicity (Western/non-Western) and by ethnic group [18–22]. Data on socio-economic status (SES) was available on a 4-digit zipcode level by open access by the Netherlands Institute for Social Research (SRP) for the year 2010. The SES was based on four indicators: the proportion of residents with a poor education level, the average household income, the proportion of residents with a low income and the proportion of unemployed residents. Ethnicity was assigned by the care giver. Western ethnicity are Dutch or other European women and non-Western are Turkish/ Moroccan, African, South Asian or other non-Western women [19]. The obstetric system in the Netherlands is characterized by a formal distinction between independent midwife-led care or primary care and obstetrician-led care or secondary care. Pregnant women without risk factors are in midwife-led care, if complications occur or risk factors are identified, women are referred to obstetrician-led care. Women at term who start labour in midwife-led care can opt for midwife-led hospital birth or homebirth [25]. For (post) term birth the line (primary/secondary) of care at start of labour and at birth is analysed.

### Statistical analysis

To investigate whether there was a trend between 2010 and 2015 among the outcome measures, first we performed a Cochran-Armitage Trend test with year of birth as the independent variable. For the main outcome measurement, we calculated adjusted odds ratios (aOR) with a 95% confidence interval (95%CI) with a multivariate logistic regression model with year of birth as the independent variable. We adjusted the year trend for general known factors that has an influence on adverse perinatal outcomes: parity, maternal age, socio-economic status and ethnicity.

The risk factors missing values were low (< 1%) and these were with single imputation assigned to the following groups: missing maternal age (19 records) was imputed with the mean age of 30 years. Missing parity (45 records) was assigned to nulliparity, 5139 records (0.6%) with missing zip code were assigned to the mid SES and 7.188 records (0.9%) with unknown ethnicity were coded to non-Western ethnicity.

The absolute difference between the first and last year is the difference between the year 2010 and the year 2015 is calculated. Because there were less births in 2015 we also calculated a standardized difference where the numbers of birth in 2015 were standardized (the number of births in 2015 divided by the number of births in 2010).

We analyse the main trend in perinatal mortality by six gestational age groups. The crude and adjusted risk of parity, maternal age, socio-economic factor and ethnicity on perinatal mortality is calculated. The trend in perinatal mortality risk among the intermediate risk groups, among the risk factors and by line of care of the six years is analysed and tested with a trend test. Statistical significance was set at the 0.05 level. Data were analysed using SAS software (SAS version 9.4; SAS Institute Cary, North Carolina, USA).

## Results

**Fig. 1:**
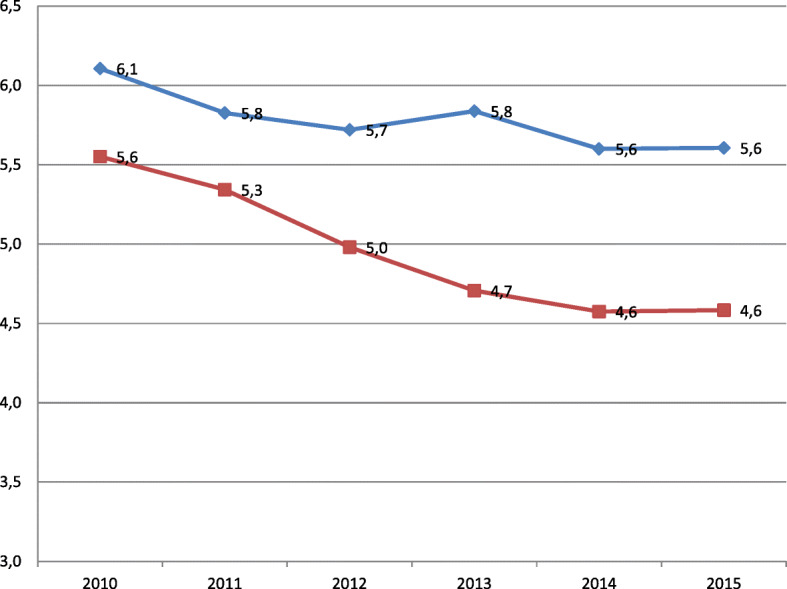
Trend in preterm birth rate per 100 births and perinatal mortality rate per 1000 birth, 2010–2015. Perinatal mortality rate per 1000 births (red line). Preterm birth rate per 100 births (blue line)

The overall perinatal mortality from 24 weeks onwards was 5.0 per 1000 births. Perinatal mortality significantly declined with 18% from 5.6 in 2010 to 4.6 per 1000 in 2015 (*p* < 0.0001) (Table 1). The adjusted Odds Ratios (aOR) of year for perinatal mortality was significantly reduced with 4% between 2010 and 2015, 0.96, 95% confidence interval (CI) [0.95–0.98] (Supplement Table 1, Fig. 1).

**Table 1:** Trend in perinatal mortality, stillbirths and neonatal mortality from 24 weeks onwards in the Netherlands between 2010 and 2015

Year	Total births	Perinatal mortality		Stillbirth			Neonatal mortality	
						Live born		
	N	n	per 1000	n	Per 1000	n	n	Per 1000
2010	170,943	949	5.55	616	3.60	170,327	333	1.96
2011	170,491	911	5.34	592	3.47	169,899	319	1.88
2012	167,669	835	4.98	552	3.29	167,117	283	1.69
2013	160,619	756	4.71	470	2.93	160,149	286	1.79
2014	165,259	756	4.57	451	2.73	164,808	305	1.85
2015	161,442	740	4.58	461	2.86	160,981	279	1.73
Total	996,423	4947	4.96	3142	3.15	993,281	1.805	1.82
Decline 2015–2010 (n)		−209		− 155			−54	
Percentage change			−18%		− 21%			− 12%
Cochran Armitage trend test (*p*-value)			< 0.0001		< 0.0001			0.0005

Stillbirth also significantly decreased with 21% from 3.6 in 2010 to 2.9 per 1000 in 2015 (*p* < 0.0001, aOR 0.95, 95%CI [0.93–0.97]). Neonatal mortality significantly decreased with 12% from 2.0 in 2010 to 1.7 per 1000 (*p* = 0.0005). The aOR of neonatal mortality was significantly decreased with 0.96 95%CI [0.93–0.98] (Table 1 and Supplement Table 1).

Compared to the 949 deaths in year 2010 there were 209 less perinatal mortality cases in the year 2015. Most of the reduction (74%) of the perinatal deaths was seen in the stillborns (Table 1).

The overall prevalence of preterm birth < 37 was 5.8% (57,647/996,423) and the prevalence of preterm birth significantly declined with 8% from 6.1 to 5.6%, p < 0.0001 (Table 2, Fig. 1). Among the preterm borns in the year 2015 compared to the year 2010 there were 115 less perinatal mortalities. Among the preterm borns there was a significant perinatal mortality risk reduction from 58 to 54 per 1000 (*p* = 0.04) (Table 2).

**Table 2:** Trend in preterm birth, congenital anomalies and SGA rates and perinatal mortality risk rates in these three intermediate risk groups

Year	N	Preterm births				Congenital anomalies				SGAp10			
		Rates		mortality risk		Rates		mortality risk		Rates		mortality risk	
		N	%	n	Per 1000	n	%	n	Per 1000	n	%	n	Per 1000
2010	170,943	10,439	6.1	601	58	4833	2.8	216	45	14,765	8.6	284	19
2011	170,491	9934	5.8	552	56	5489	3.2	199	36	14,785	8.7	260	18
2012	167,669	9591	5.7	547	57	4473	2.7	186	42	14,382	8.6	244	17
2013	160,619	9377	5.8	484	52	4902	3.1	182	37	13,880	8.6	209	15
2014	165,259	9255	5.6	471	51	4660	2.8	175	38	14,172	8.6	222	16
2015	161,442	9051	5.6	486	54	4479	2.8	177	40	13,925	8.6	212	15
Total	996,423	57,647	5.8	3141	54	28,836	2.9	415	39	85,909	8.6	1431	17
Percentage change 2015–2010 (%)			−8%				−2%				0%		
Trend test (p-value) < 0.0001							0.61				0.17		
Trend test mortality risk (p-value)					0.0418				0.002				0.006
Difference in mortality 2015–2010 (n)				−115				−39				−72	

Table 3 shows the trend of perinatal mortality by gestational age groups. The largest decrease in perinatal mortality risk reduction of 32% was seen between 24 and 27 weeks of gestation, the risk declined from 497 in 2010 to 339 per 1000 in 2015, *p* < 0.001 (55 deaths). Between 28 and 31 weeks the reduction in perinatal mortality was 12%, from 139 in 2010 to 123 per 1000 in 2015 (26 deaths). The smallest reduction was seen between 32 and 36 weeks of gestation with a 3% reduction (34 deaths).

**Table 3:** Difference in perinatal mortality between 2010 and 2015 by gestational age groups

2010–2015		2010–15	2010	2015	2010	2015	Relative	Absolute	Stand.
gestational		Total births	perinatal mortality				Difference		
weeks		n	n	n	per 1000	per 1000	%	n	n
**24.0–27.6**		**3079**	**256**	**201**	**497**	**339**	**−32%**	**−55**	**−70**
**28.0–31.6**		**5835**	**141**	**115**	**139**	**123**	**−12%**	**−26**	**−34**
**32.0–36.6**		**48,733**	**204**	**170**	**22.9**	**22.3**	**−3%**	**−34**	**−46**
**37.0–42.6**		**938,776**	**348**	**254**	**2.2**	**1.7**	**−23%**	**−94**	**−115**
	37.0–38.6	223,097	147	109	3.9	2.9	− 26%	−38	−47
	39.0–40.6	528,405	145	108	1.6	1.2	−25%	−37	− 46
	41.0–42.6	187,274	56	37	1.7	1.3	−24%	−19	− 22
**Total**		**996,423**	**949**	**740**	**5.6**	**4.6**	**18%**	**−209**	**−265**

*Stand*. Standardized

In the term and post term population, perinatal mortality risk is low (2.2 in 2010 and 1.7 per 1000 in 2015) and the reduction in perinatal mortality was 23% (94 deaths). In the 37–38 weeks of gestation perinatal mortality declined from 3.9 in 2010 to 2.9 per 1000 in 2015 (38 deaths). In the 39–40 weeks perinatal mortality declined as in the 41–42 weeks (Table 3). Since the overall number of births declined slightly in 2015 we standardized the 2015 numbers and the standardized reduction in perinatal mortality between the year 2010 and 2015 was 265 deaths (Table 3).

The prevalence of congenital anomalies was 2.9% and did not change much between 2010 and 2015 (Table 2). Among the children with congenital anomalies, there was a significant decline in perinatal mortality risks (45 in 2010 to 40 per 1000 in 2015). There was a reduction of 39 deaths.

The prevalence of SGAp10 was 8.6% (Table 2). There was no trend in the incidence of SGAp10. A significant decline in perinatal mortality risk was seen in SGA children (19 in 2010 to 15 per 1000 in 2015, *p* = 0.006) (Table 2). There was a reduction of 72 deaths.

### Risk factors

In Table 4 the crude and adjusted risks for perinatal mortality are shown. The overall mortality risk of 5.0 per 1000 is about as twice as high for women with an African ethnicity (10.1 per 1000), and for women with a maternal age ≥ 40 years (9.0 per 1000). Women with parity 2 or higher and women with low socio-economic status, have also a significantly increased risk of 5.9 per 1000 (Table 4).

**Table 4:** The incidence of risk factors and perinatal mortality risk

Risk factors		Perinatal mortality per 1000 births						
	N	Per 1000	Crude Odds		Adjusted odds *1		Adjusted odds *2	
Total	4947	5.0		(95% CI)		(95% CI)		(95% CI)
Year			0.96	(0.94–0.97)	0.96	(0.94–0.97)	0.96	(0.95–0.98)
Parity								
P0	45.5%	5.5	1.44	(1.35–1.54)	1.45	(1.36–1.55)	1.09	(1.01–1.17)
P1	35.7%	3.8	1.00		1.00		1.00	
P2+	18.8%	5.9	1.56	(1.44–1.69)	1.38	(1.28–1.50)	1.35	(1.24–1.47)
Maternal age (years)								
< 20	1.2%	6.5	1.43	(1.14–1.79)	1.11	(0.88–1.39)	1.04	(0.82–1.31)
20–24	10.0%	5.8	1.27	(1.16–1.39)	1.11	(1.01–1.21)	1.07	(0.97–1.17)
25–34	68.1%	4.5	1.00		1.00		1.00	
35–39	17.4%	5.3	1.16	(1.08–1.25)	1.18	(1.10–1.28)	1.07	(0.99–1.16)
> =40	3.3%	9.0	2.00	(1.77–2.25)	1.92	(1.70–2.17)	1.38	(1.21–1.57)
Ethnicity								
Dutch	75.6%	4.4	1.00		1.00		1.00	
Other European	4.8%	4.9	1.12	(0.98–1.28)	1.09	(0.95–1.24)	1.10	(0.96–1.26)
Mediterranean	7.0%	6.7	1.52	(1.38–1.68)	1.42	(1.29–1.57)	1.39	(1.25–1.55)
African	2.6%	10.1	2.30	(2.03–2.61)	2.12	(1.87–2.42)	1.50	(1.31–1.72)
South Asian	1.2%	6.0	1.37	(1.08–1.72)	1.34	(1.06–1.69)	0.76	(0.60–0.97)
Asian	2.6%	5.3	1.19	(1.01–1.42)	1.17	(0.99–1.39)	1.08	(0.90–1.29)
other non-Western	6.0%	7.4	1.69	(1.53–1.87)	1.63	(1.47–1.80)	1.47	(1.32–1.63)
Social-Economic status								
Low	24.7%	5.9	1.21	(1.13–1.29)	1.06	(0.99–1.14)	1.00	(0.93–1.08)
Mid	50.5%	4.9	1.00		1.00		1.00	
High	24.8%	4.2	0.86	(0.80–0.93)	0.86	(0.80–0.92)	0.92	(0.86–0.997)
**Intermediate risk factors**								
Preterm birth < 37 weeks	6.1%	95.3	53.3	(50.3–56.5)			25.8	(24.3–27.4)
Congenital anomalies	3.1%	83.4	17.2	(16.3–18.1)			5.2	(4.8–5.6)
Small gestational age (SGA) P10	8.8%	38.8	8.68	(8.26–9.12)			3.9	(3.7–4.2)

Adjusted for:

*1 Age, parity, ethnicity en SES

*2 = Age, parity, ethnicity, SES, prematurity, congenital anomalies and SGA

After adjustment African ethnicity has a significant increased adjusted odds ratio (aOR) for perinatal mortality of 2.1 95%CI (1.9–2.4). Also, high maternal age ≥ 40 years with aOR 1.9 95%CI (1.7–2.2) and parity 2+ with aOR 1.4 95%CI (1.3–1.5) are still significant risk factors for perinatal mortality, while low maternal age and low SES are no longer significant risk factors for perinatal mortality. African ethnicity, maternal age 40^+^ and parity 2^+^ were still significant risk factors after further adjustment for the intermediate factors including preterm birth (Table 4).

Both in the high and low risk groups perinatal mortality declined between 2010 and 2015 (Table 5). The strongest significant reduction (195 deaths) from 5.0 in 2010 to 4.1 per 1000 in 2015 was seen in the largest groups; the children of Western women (p = < 0.0001). Among the nulliparous women also perinatal mortality decreased (124 deaths) from 5.9 in 2010 to 5.0 per 1000 in 2015, *p* = 0.0011. And among women with an average maternal age of 25 to 34 years perinatal mortality significantly reduced with 85 deaths from 5.0 in 2010 to 4.3 per 1000 in 2015 (*p* = 0.0024).

**Table 5:** The perinatal mortality risk per 1000 births by year in risk groups

Year	P0	P2+	< 20 yrs	25–34 yrs	≥40 yrs	low SES	High SES	Western ethnicity	non-Western ethnicity
2010	5.9	6.4	7.4	5.0	9.5	7.1	4.4	5.0	8.1
2011	6.1	6.0	8.9	4.8	9.0	5.5	4.8	4.9	7.0
2012	5.3	6.3	6.9	4.4	11.8	6.1	4.2	4.2	7.8
2013	5.4	6.2	3.2	4.4	8.2	6.0	4.0	4.3	6.3
2014	5.1	5.3	6.9	4.3	7.2	5.6	3.8	3.9	7.2
2015	5.0	5.3	4.5	4.3	8.3	5.1	4.1	4.1	6.4
Total	5.5	5.9	6.5	4.5	9.0	5.9	4.2	4.4	7.1
Total N	2487	1109	79	3084	296	1448	1040	3558	948
Diff 2010–15	−124	−29	−11	−85	−11	−86	−31	−195	−56
Trend test	0.0011	0.0268	0.10	0.0024	0.15	0.028	0.0599	< 0.0001	0.02

*Diff* difference in number of children with perinatal mortality between year 2010 and year 2015

*Yrs* years

The overall decline in perinatal mortality was seen in other high risk groups although not always significant. The trend significantly declined among all non-Western women (8.1 in 2010 to 6.4 per 1000 in 2015, *p* = 0.02) and among the women with low SES (7.1 to 5.1 per 1000, *p* = 0.028). In the smaller groups, the decline was not significant: the women with the age of 40 years or higher the perinatal mortality risk was high 9.5 per 1000 and declined to 8.3 per 1000 (*p* = 0.15) and among the women with low maternal age (< 20) risk declined from 7.4 to 4.5 per 1000, *p* = 0.10 (Table 5).

Among the (post)term women there was no significant decline in perinatal mortality in women with low (< 25 year) maternal age, in women with low and high SES and in women with non-Western ethnicity (Table 6). Also in (post)term women perinatal mortality risk did not significantly decline in women delivered under the supervision of a midwife (primary care) (0.52 in 2010 to 0.35 per 1000 in 2015, *p* = 0.45, 9 deaths). However perinatal mortality significantly declined in (post)term women delivered under supervision of an obstetrician (secondary care) (2.9 in 2010 to 2.2 per 1000 in 2015, *p* < 0.0001, 85 deaths). Perinatal mortality risks at start of labour showed the same pattern; a significant reduction in perinatal mortality in women who started delivery in secondary care but not in women who started delivery in primary care (Table 6).

**Table 6:** The perinatal mortality risk per 1000 births by year in risk groups in (post)term women

Year	P0	P2+	< 25 yr	25–34 yrs	> = 35 yrs	low SES	High SES	Western ethnicity	non-Western ethnicity	Primary care start labour	Secondary care start labour	Primary care at birth	Secon- dary care at birth	Total
2010	2.3	2.6	2.0	2.1	2.6	2.7	1.8	2.0	2.9	1.0	3.5	0.52	2.9	2.2
2011	2.4	2.6	2.0	2.1	2.9	1.7	2.1	2.2	2.5	1.0	3.5	0.63	2.9	2.2
2012	1.9	2.6	1.8	1.7	2.3	2.1	1.5	1.7	2.5	0.8	3.0	0.40	2.5	1.8
2013	2.1	2.5	1.8	1.7	2.1	2.1	1.5	1.6	2.4	0.9	2.8	0.40	2.4	1.8
2014	2.2	1.7	1.7	1.8	2.0	2.0	1.6	1.6	2.7	1.0	2.8	0.69	2.3	1.8
2015	1.7	1.8	1.8	1.5	2.1	1.8	1.5	1.5	2.4	0.8	2.7	0.35	2.2	1.7
Total	2.1	2.3	1.8	1.8	2.3	2.1	1.7	1.8	2.6	0.9	3.0	0.50	2.5	1.9
Total N	889	411	192	1158	456	480	389	1343	463	466	1319	142	1664	1806
Diff 10–15	− 62	−20	−9	−30	−24	−36	−16	−89	−5	−24	−73	−9	−85	−94
Trend test	0.016	0.0029	0.53	0.0011	0.0029	0.077	0.08	< 0.0001	0.38	0.17	0.0001	0.45	< 0.0001	< 0.0001

*Diff 10–15* difference in number of children with perinatal mortality between 2010 and 2015

## Discussion

### Main findings

In the Netherlands, perinatal mortality has significantly declined with 18% and preterm birth has significantly declined with 8% between 2010 and 2015. The largest decline in perinatal mortality risk of 32% is among the children born between 24 and 27 weeks of gestation. Among 37–42 weeks of gestation mortality risk reduction was 23%. Perinatal mortality risk significantly declined among all intermediate risk groups; the children with preterm birth, SGA and/or congenital anomalies. In addition, the main risk factors for perinatal mortality were African ethnicity, maternal age ≥ 40 years and parity 2^+^. In the largest groups like nulliparity, low SES and non-Western ethnicity a significant decline in perinatal mortality is seen. On the contrary, at term, there was no significant decline in perinatal mortality in women with low age, in women with low or high SES, in women with non-Western ethnicity and among women who started or delivered under primary care.

### Strengths and limitations

A strength of our study is that results are based on data obtained from the national perinatal registry databases with almost a million records, which are nearly complete (98% of all births in the Netherlands) including many risk factors. For trend analysis of perinatal mortality outcomes, large and complete databases are needed.

A potential limitation could be the exclusion of births below 24 weeks and above 43 weeks of gestation. However, among the children born during 22.0–23.6 weeks of gestation there is also a significant decline in perinatal mortality from 970 per 1000 in 2010 to 929 per 1000 in 2015. The effect of excluding post term births ≥43.0 weeks on the analysis was very low because there were only 54 women in this group.

The number of missing values of the risk factors is very low (< 1%). However, excluding missing values from the analysis showed also a similar main result: a significant declining trend in preterm birth and in perinatal mortality. A limitation of the national perinatal registry is the lack of information on smoking during gestation and maternal obesity (BMI). Trends among these important risk groups could not be provided.

### Comparison with the literature

Studies on recent overall trends in perinatal mortality from 2010 onwards in other European countries are published [4, 7, 27], as is the latest European perinatal health report of 2015 [5]. These show declining trends in perinatal mortality in different European countries; stillbirth rates in 2015 were 5% lower than in 2010, but this reflects an average with large differences between the countries. The European Peristat report also showed that for the Netherlands stillbirth rates after 28 weeks, including multiple births, declined from 2.9 in 2010 to 2.2 per 1000 in 2015.

In addition, the neonatal mortality declined in some countries in Europe. However in Poland neonatal mortality rates increased. In the Netherlands neonatal mortality rate after 24 weeks of gestation, including multiple births, declined from 2.2 in 2010 to 2.0 per 1000 in 2015 [5]. In contrast to Peristat, which is based on aggregated date, the more detailed studies on perinatal mortality risk among different (intermediate) risk groups such as in our study is unique. Termination of pregnancy (TOP) still forms a substantial proportion of the stillbirths at 22 and 23 weeks of gestation. In the Netherlands TOP is not registered as a separate group in the national registry. Iatrogenic start of labour accounts in total 300 (66%) of the 458 perinatal deaths at 22–23 weeks in 2010. To make international comparisons of stillbirths rates at 22 and 23 weeks possible in the Netherlands TOP’s should be registered and only stillbirths without TOP should be included in datasets for international /European comparison [4, 28]. In this study, the threshold of 24 weeks of gestation for both stillbirth and neonatal mortality was used. This is in line with the recent Lancet publication of the PERISTAT researchers [4]. There it is stated, that the threshold of 28 weeks for international comparisons should be lowered to 24 weeks for high-income countries acknowledging the burden of perinatal death to families. Current international comparisons exclude a third of all stillbirths [4].

### Interpretation/clinical implications and future research

Various interventions for reducing perinatal mortality in the Netherlands have taken place. The national perinatal audit (PAN) resulted in a description of substandard factors in relation to death and the recommendation for better care management, cooperation, communication and guidelines, ready for implementation [9]. The audit also resulted in better cooperation between care providers in local and regional settings and this may have been effective in reducing perinatal mortality.

Active neonatal treatment starting at 24 weeks of gestation resulted in significant improvement in neonatal survival [8]. The significant decline in perinatal mortality among SGA children suggests better detection and surveillance. In general, the decline in perinatal mortality is mainly based on the reduction of stillbirths. The 32% decline in perinatal mortality among the children born between 24 and 27 weeks of gestation (55 deaths) is probably due to changes in 2010 in the 3rd level care; there is more active management at 24 weeks of gestation [29]. The prevention of stillbirth mortality probably was the result of earlier intervention by obstetricians and more active intensive neonatal care of the newborn. In the children born between 24.0 and 26.6 weeks there were significantly more interventions; the total caesarean sections rate in this subgroup increased from 27.6 to 35.9% and prelabour caesarean sections increased from 19.4 to 25.4% (data not shown).

In the Netherlands between 2010 and 2015, the number of preterm births < 37 weeks declined with 8% from 6.1 to 5.6% and this could be one of the explanations of the lower total perinatal mortality rates. In some other European countries, also preterm birth rates are declining but in some other countries preterm birth rates have increased. Prevention of spontaneous preterm birth stays a permanent goal in obstetric care. There was only a 12% reduction in perinatal mortality (26 deaths) between 28 and 31 weeks of gestation and 3% reduction (34 deaths) between 32 and 36 weeks of gestation. Possibly, there is still room for further reduction of perinatal mortality in these late preterm gestational age groups. The 23% perinatal mortality reduction among 37–42 weeks of gestation is of importance since this is the largest group (94%) of all births. The national perinatal audit, which started in 2010, has focused in these years on term pregnancy mortality [9]. National audit programs should be implemented in other high-income countries to further reduce perinatal mortalities. Deliveries of 42 weeks or more, which is a risk factor for perinatal mortality, is reduced in the Netherlands by more referral and active management [12, 30].

Since 2010, the number of deliveries under supervision of a midwife is reduced. More women are transferred during pregnancy or during childbirth from 1st to 2nd level of care. The risk of perinatal mortality among the (post) term women delivered under supervision of a midwife is low (0.5 per 1000) and declined, however not significantly. However, the perinatal mortality risk among the women delivered under supervision of an obstetrician significantly declined. Maybe the current implementation of the recently developed standard of integrated care can make the decline continue.

Perinatal mortality among the children with congenital anomalies and in the children with SGA has been reduced. The introduction of the 20 week structural echo may have helped in the detection of congenital anomalies [11]. Detection of SGA during gestation still forms a challenge.

Risk factors for perinatal mortality, preterm birth and small for gestational age (SGA) are cigarette smoking, maternal age (< 20, ≥ 40 years), non-Western ethnicity, genito-urinary tract infections, poor nutrition and psychological stress related to economic disadvantage. Some of these factors are modifiable by better health care management, education or public health intervention(s). Nevertheless, many biological factors are more difficult to improve.

A possible target for reduction of perinatal mortality could be deliveries among 28–36 weeks of gestation. In future studies, trends in different risk groups should be monitored and effort has to be made in order to obtain comparable national registry data over the years, including information on termination of pregnancy, smoking during gestation and on body mass index.

## Conclusions

There is a decline in perinatal mortality and in preterm births between 2010 and 2015. The decline of perinatal mortality is most prominent among 24–27 weeks of gestation and among (post)term births. Perinatal mortality reduction in the gestational age group among 32–36 weeks, in women with high age, non-Western ethnicity and (post) term women who started or delivered under primary care is a focus point for awareness and further research.

## Supplementary information

**Additional file 1: Table 1**. Odd’s ratio’s of trend in perinatal mortality, stillbirth and neonatal mortality.

## Data Availability

There is a formal request policy for permission and the use of the national perinatal registry “PERINED” data in the Netherlands (www.perined.nl). More information is available from on the website of PERINED (https://www.perined.nl/voor-wie-werken-we/onderzoek-aanvragen). Available public information: each year the national registry PERINED makes a summary of the main data available: for the year 2010 https://assets.perined.nl/docs/d5e0359b-6030-4b01-b40b-0c0dc6959b19.PDF for 2011: https://assets.perined.nl/docs/ec7434e3-c899-4008-8a04-b3068f285b86.PDFfor 2012: https://assets.perined.nl/docs/41346c24-0a52-4a26-9544-7cb3b7d78cc0.PDFfor 2013: https://perined-assets.e-dev.nl/docs/3a1a6fe5-08aa-455f-8f43-77a176f95f79.pdffor 2014: https://assets.perined.nl/docs/353d9249-9875-4cb3-9c86-f078ae3f7aef.pdf for 2015: https://assets.perined.nl/docs/980021f9-6364-4dc1-9147-d976d6f4af8c.pdf.

## Abbreviations

NICU: Neonatal Intensive Care Unit
OR: Odds Ratio
PRN: Perinatal Registry Netherlands (now PERINED)
PERINED: Perinatal Registry Netherlands (formal named PRN)
SGA: Small for gestational Age
SES: Socio Economic Status
