# Supplementary material for: Decreasing trend in preterm birth and perinatal mortality, do disparities also decline?
Source: BMC Public Health. 2020 May 26;20:783. doi: 10.1186/s12889-020-08925-w (PMC7249399; doi:10.1186/s12889-020-08925-w)
Supplement: Supplementary file 1 — Additional file 1: Table 1. Odd’s ratio’s of trend in perinatal mortality, stillbirth and neonatal mortality. [file 12889_2020_8925_MOESM1_ESM.docx]

**Supplement Table 1**

| **Odds ratios of trend in perinatal mortality, stillbirth and neonatal mortality.** | | | | | |  |  |
| --- | --- | --- | --- | --- | --- | --- | --- |
|  |  |  |  |  |  |  |  |
|  |  | **Perinatal mortality** | | **Stillbirth** | | **Neonatal mortality** | |
|  |  | **Odds (95% CI)** | | **Odds (95% CI)** | | **Odds (95% CI)** | |
| **crude year** |  | 0.96 | (0.94-0.97) | 0.94 | (0.92-0.96) | 0.95 | (0.93-0.98) |
| **adjusted *1** |  | 0.96 | (0.94-0.97) | 0.94 | (0.92-0.96) | 0.95 | (0.93-0.98) |
| **adjusted *2** |  | 0.96 | (0.95-0.98) | 0.95 | (0.93-0.97) | 0.96 | (0.93-0.98) |
|  |  |  |  |  |  |  |  |
| *1= adjusted for Age, parity, ethnicity and SES | | | |  |  |  |  |
| *2= adjusted for Age, parity, ethnicity, SES, prematurity, congenital anomalies and SGA | | | | | | |  |
